# Cellulose-Based Hydrogels Incorporating Olive Mill Wastewater (OMW): Preparation, Characterization, and In Vitro Antimicrobial Activity

**DOI:** 10.3390/gels12040282

**Published:** 2026-03-27

**Authors:** Eleonora Russo, Debora Caviglia, Anna Maria Schito, Carla Villa

**Affiliations:** 1 Department of Pharmacy, University of Genova, Viale Benedetto XV, 3, 16132 Genoa, Italy; debora.caviglia@edu.unige.it (D.C.); carla.villa@unige.it (C.V.); 2 Department of Integrated Surgical and Diagnostic Sciences, University of Genova, Viale Benedetto XV, 6, 16132 Genoa, Italy; anna.maria.schito@unige.it

**Keywords:** cellulose hydrogels, polyphenols, antioxidant activity, antimicrobial activity, topical formulation, drug delivery

## Abstract

Olive mill wastewater (OMW) is an agro-industrial byproduct rich in polyphenols and other bioactive compounds with documented antioxidant and antimicrobial properties. In this study, purified OMW fractions (RO1 and MD2), previously characterized by high polyphenol content and strong antioxidant activity, were incorporated (10% *w*/*w*) into cellulose-based hydrogels intended for topical application. Hydrogels were prepared using carboxymethyl cellulose (CMC), hydroxyethyl cellulose (HEC), hydroxypropyl methylcellulose (HPMC), and methylcellulose (MC) at concentrations of 1.5–2.0% (*w*/*w*). The formulations were characterized in terms of organoleptic properties, pH, rheological behavior, swelling capacity, weight loss, antioxidant activity (DPPH assay), and microbiological activity against selected skin pathogens, including antibiotic-resistant strains. Rheological analysis confirmed pseudoplastic behavior suitable for topical administration. OMW-loaded hydrogels exhibited significant radical scavenging activity compared to blank formulations and demonstrated antimicrobial efficacy, supporting the preservation of OMW bioactivity within the polymeric network. The results highlight the potential of cellulose-based hydrogels as sustainable and biocompatible carriers for the valorization of OMW in dermatological applications, particularly for the management of oxidative stress and bacterial skin infections.

## 1. Introduction

Olive mill wastewater (OMW) is an aqueous byproduct generated during the extraction of olive oil and consists of water, organic matter, and a variety of bioactive compounds such as polyphenols, flavonoids, and phenolic acids. Traditionally, OMW has been regarded as an environmental pollutant, with its high chemical oxygen demand (COD), phytotoxicity, and phenolic content making it difficult to manage and dispose of sustainably. However, recent advances in waste valorization and circular economy strategies have shifted the focus toward utilizing OMW as a valuable resource [1] for the development of novel bioactive materials [2]. In this context, OMW is increasingly considered a rich source of natural antioxidants and multifunctional phenolic compounds with potential biomedical applications [3]. Moreover, spray-dried OMW-based systems have demonstrated significant antioxidant and wound healing potential, further supporting the feasibility of transforming OMW into high-value biomedical materials [4].

Recent studies on purified OMW extracts have shown anti-angiogenic and chemopreventive in vitro and in vivo effects, as well as inhibition of endothelial cell proliferation, migration, and invasion [5,6,7]. OMW has also demonstrated antiproliferative activity against MDA-MB-231 breast cancer cells [8], as well as chemopreventive effects in leukemia and colon cancer cells through apoptosis induction [9]. These findings suggest that the biological activity of OMW is strongly associated with its phenolic composition, particularly the hydroxytyrosol (HT), tyrosol (T), and oleuropein (OL) derivatives. OMW is known to contain a complex mixture of phenolic compounds, and other secoiridoids represent the major bioactive constituents. These molecules are widely recognized for their strong antioxidant and antimicrobial properties and are considered responsible for most of the biological activities associated with OMW-derived extracts.

OMW extracts exhibit neuroprotective in vitro and in vivo effects on brain cells from NMRI mice, likely due to their antioxidant and anti-inflammatory properties. Polyphenols in OMW help delay cellular aging in neurodegenerative diseases by preventing aggregation of amylin, tau, and Aβ peptides [10,11,12]. Biophenols also reduce oxidative stress in neuroblastoma cells [13], further supporting their potential in mitigating oxidative stress-related disorders.

OMW formulations have shown cytoprotective effects against heavy metals like cadmium, mercury, and lead [14], and their phenolic compounds may reduce risks for coronary heart disease and stroke [15]. Furthermore, tyrosol, hydroxytyrosol and oleuropein help mitigate the effects of high-fat/sugar diets by regulating nitric oxide and endothelin-1 (ET-1) levels, thereby improving endothelial function in diabetic conditions [16]. Collectively, these data highlight the multifunctional biological profile of OMW-derived fractions, including their antioxidant, anti-inflammatory, cardioprotective, and antimicrobial activities.

Despite these promising properties, the practical application of OMW in biomedical contexts requires appropriate delivery systems that are capable of stabilizing and preserving its bioactive components while ensuring controlled and localized release. One promising approach is the formulation of OMW into hydrogels, three-dimensional, water-swollen polymeric networks capable of retaining large amounts of water. Hydrogels are biocompatible, non-toxic [17], and capable of sustained release, making them ideal materials for a range of biomedical applications [18]. These include wound healing [19], drug delivery [20], and topical treatments, particularly for skin diseases. Their high water content, structural similarity to the extracellular matrix, and ability to incorporate both hydrophilic and moderately hydrophobic compounds make hydrogels especially attractive for dermal and transdermal applications.

Hydrogels can be formulated from a variety of natural and synthetic polymers, among which biopolymers such as cellulose derivatives are particularly advantageous due to their safety profile and regulatory acceptance in pharmaceutical formulations [21,22]. Cellulose, a biopolymer derived from plant cell walls, is widely employed in the preparation of pharmaceutical hydrogels owing to its abundance, biodegradability, biocompatibility, and chemical versatility. Cellulose and its derivatives, such as carboxymethylcellulose (CMC), hydroxypropyl cellulose (HPC), methylcellulose (MC), and hydroxyethyl cellulose (HEC), exhibit excellent water retention capability, swelling capacity, and rheological properties, making them ideal candidates for hydrogel formulations [23].

Cellulose-based hydrogels can be prepared using different crosslinking strategies, including chemical, physical, and enzymatic approaches. These strategies allow the modulation of mechanical strength, porosity, swelling behavior, and drug release kinetics [24]. The tunability of cellulose networks enables the design of hydrogels with tailored properties suitable for specific biomedical purposes.

One of the key advantages of cellulose-based hydrogels is their potential for site-specific drug delivery. Hydrogels can be engineered to respond to environmental stimuli [25] such as pH, temperature, ionic strength, or enzymatic activity, enabling controlled and targeted release of therapeutic agents. The ease of chemical modification and functionalization of cellulose enhances its adaptability to various pharmaceutical applications, including ocular, transdermal, and oral drug delivery systems [26,27].

Recent research has focused on improving the mechanical stability, bioadhesion, and structural integrity of cellulose-based hydrogels while maintaining their biocompatibility and biodegradability [28], with the aim of optimizing their performance in wound management and antimicrobial therapies.

In a previously published paper [29], two purified OMW fractions, designated as RO1 and MD2, were identified as particularly microbiologically active, exhibiting strong antioxidant activity (*AA*%) and the highest polyphenol content. For consistency, the same nomenclature (RO1 and MD2) is retained in the present study.

Given their bioactive profile, RO1, MD2, and their combination (COMBO) were incorporated into cellulose-based hydrogels (CMC, HEC, HPMC, and MC) with the aim of exploring their potential for topical application in the treatment of epidermal and dermal bacterial infections, particularly those caused by antibiotic-resistant pathogens [30].

It is important to emphasize that the antimicrobial activity observed for the purified OMW fractions was preserved in the corresponding hydrogel formulations, confirming that incorporation into the polymeric network did not compromise their biological efficacy. These findings suggest that cellulose-based hydrogels may represent promising carriers for OMW-derived bioactive fractions in topical antimicrobial therapy [31].

## 2. Results and Discussion

### 2.1. Physicochemical and Technological Characterization of Hydrogels

Preliminary evaluations indicated that the incorporation of OMW fractions did not significantly affect the main physicochemical properties of the hydrogel matrices (pH, rheology, swelling, and weight loss). Therefore, the characterization results based on the hydrogel systems are reported, while the biological activities are discussed specifically for the OMW-loaded formulations.

#### 2.1.1. Organoleptic Evaluation

**Figure 1:**
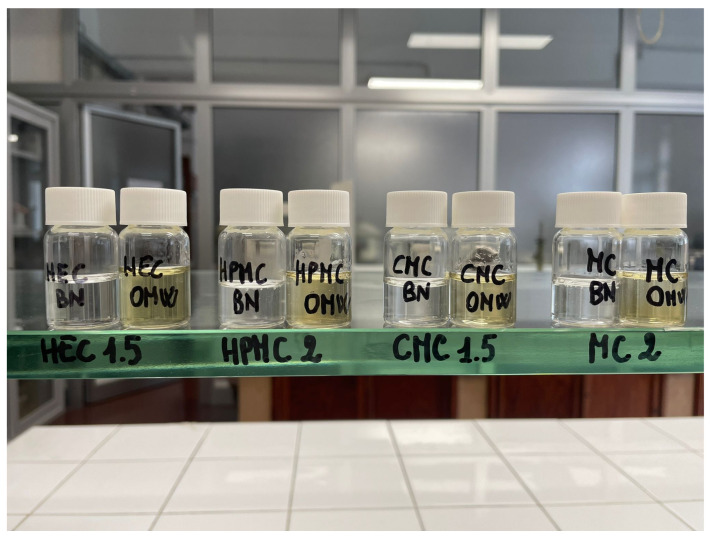
Appearance of all cellulose hydrogels: blank and the hydrogel containing 10% (*w*/*w*) olive mill wastewater (OMW).

All hydrogels appeared homogeneous and free of visible polymer aggregates or phase separation immediately after preparation. The blank formulations were transparent and colorless, exhibiting a uniform gel structure without air bubbles. In contrast, the hydrogels containing 10% (*w*/*w*) OMW showed a characteristic yellow coloration attributable to the presence of polyphenolic compounds (Figure 1). Despite the color change, the OMW-loaded hydrogel remained macroscopically homogeneous, with no evidence of syneresis or phase separation during the observation period. The incorporation of OMW did not adversely affect the structural integrity or visual appearance of the hydrogel matrix.

#### 2.1.2. pH Results Data

The pH values of the OMW-loaded cellulose-based hydrogels are reported in Table 1.

All formulations exhibited slightly acidic pH values ranging from 4.45 to 4.97. Hydrogels containing the MD2 fraction showed pH values between 4.78 and 4.97, whereas RO1-based formulations presented slightly lower values, ranging from 4.45 to 4.75. Among the tested polymers, MD2-CMC-1.5 exhibited the highest value (4.97), followed by MD2-HPMC-2 (4.95) while RO1-MC-2 showed the lowest one (4.45). In general, for each cellulose derivative (HPMC, CMC, HEC, and MC), RO1-containing hydrogels consistently displayed lower pH values compared to the corresponding MD2 formulations. The slightly acidic pH observed for all formulations is consistent with the presence of ionizable phenolic compounds and organic acids, which are naturally present in OMW. The measured pH values (4.45–4.97) fall within or close to the physiological skin pH range (approximately 4.5–5.5), indicating suitability for topical application without risks of significant skin irritation. The type of cellulose derivative did not markedly influence the pH, as differences between polymers within the same OMW fraction were minimal (<0.3 pH units). This suggests that the pH of the formulations is primarily governed by the chemical composition of the incorporated OMW fraction rather than by the polymeric matrix. The results confirm that incorporation of OMW fractions into cellulose-based hydrogels yields formulations with skin-compatible pH values while preserving the intrinsic acidic character associated with their bioactive phenolic content.

#### 2.1.3. Rheological Results

**Figure 2:**
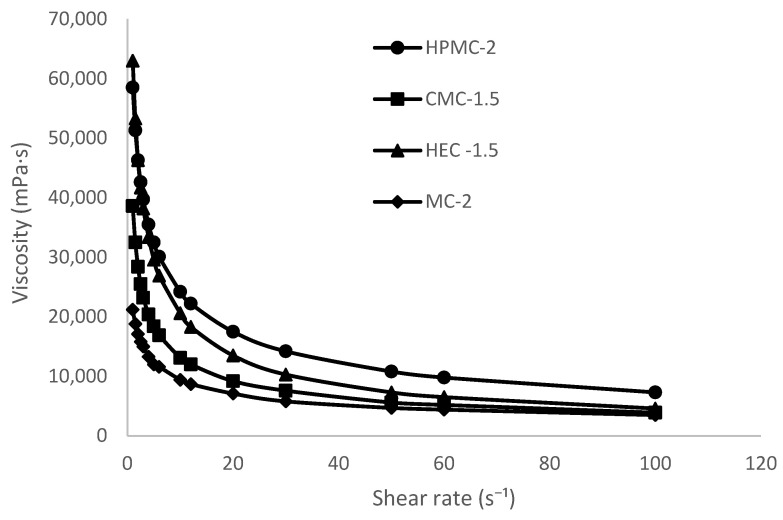
Flow curves of cellulose-based hydrogels (HPMC-2, CMC-1.5, HEC-1.5, and MC-2) showing viscosity (mPa·s) as a function of shear rate (1–100 s^−1^) at 25 ± 0.5 °C.

The flow curves of the cellulose-based hydrogels (HPMC-2, CMC-1.5, HEC-1.5, and MC-2) are shown in Figure 2. All formulations exhibited a marked decrease in viscosity with increasing shear rate, indicating a typical non-Newtonian pseudoplastic behavior.

At low shear rates (1 s^−1^), the highest viscosity was observed for HEC-1.5 (63,000 mPa·s), followed by HPMC-2 (58,000 mPa·s), CMC-1.5 (38,000 mPa·s), and MC-2 (20,000 mPa·s). As the shear rate increased to 100 s^−1^, viscosity values significantly decreased for all samples, reaching 4600 mPa·s for HEC-1.5, 7300 mPa·s for HPMC-2, 3900 mPa·s for CMC-1.5, and 3500 mPa·s for MC-2.

The pronounced shear-thinning behavior suggests progressive disruption and alignment of the polymeric network under increasing shear stress. HEC-1.5 and HPMC-2 demonstrated the highest structural consistency at low shear rates, which is indicative of a more developed three-dimensional network. Conversely, MC-2 exhibited the lowest viscosity across the entire shear range, reflecting a comparatively weaker gel structure.

The observed rheological profiles are particularly suitable for topical application: high viscosity at low shear rates ensures adequate retention on the skin surface, while reduced viscosity under shear stress facilitates spreadability during application. Overall, all formulations displayed rheological properties consistent with stable, structured hydrogel systems appropriate for dermal administration.

#### 2.1.4. Swelling Studies

**Figure 3:**
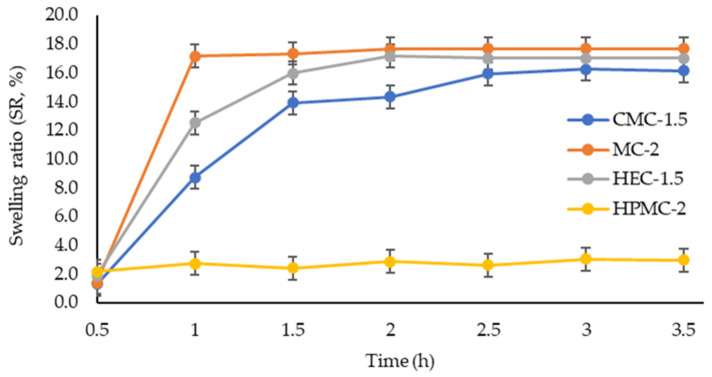
Swelling ratio (*SR*, %) of cellulose-based hydrogels (CMC-1.5, MC-2, HEC-1.5, and HPMC-2) as a function of time (0.5–3.5 h) at 25 ± 0.5 °C. Values are expressed as means ± SDs (n = 3).

The swelling profiles of the cellulose-based hydrogels (CMC-1.5, MC-2, HEC-1.5, and HPMC-2) are presented in Figure 3. The swelling ratio (*SR*, %) was evaluated over 3.5 h, and distinct behaviors were observed depending on the polymer type.

All formulations exhibited rapid initial water uptake within the first hour, followed by a gradual approach to equilibrium. MC-2 and HEC-1.5 showed the highest swelling capacity, reaching equilibrium values of approximately 17.7% and 17.0%, respectively, within 2–2.5 h. MC-2 demonstrated the fastest swelling kinetics, achieving more than 95% of its maximum swelling within the first hour (17%).

CMC-1.5 displayed a progressive increase in swelling ratio, reaching a maximum value of 16.2% at 3 h, followed by stabilization. In contrast, HPMC-2 exhibited markedly lower swelling behavior throughout the experiment, with *SR* values remaining between 2.2% and 3.0%, indicating limited water uptake capacity compared to the other cellulose derivatives. Although HPMC-based hydrogels often exhibit higher swelling values according to the literature, the lower swelling observed in the present study may be related to formulation-specific factors, including polymer–polymer interactions, network compactness, and potential interactions that occurring within the hydrogel matrix.

The rapid initial swelling phase observed for CMC, MC, and HEC can be attributed to water diffusion into the polymeric network and hydration of hydrophilic functional groups. The subsequent plateau suggests the attainment of equilibrium between osmotic forces driving water absorption and elastic retraction forces within the gel matrix. Although swelling kinetic models such as the Korsmeyer–Peppas or Fickian diffusion model are often used to describe solvent diffusion in hydrogel networks, the present study focused primarily on comparative swelling behavior among the different formulations. Overall, the swelling capacity followed the order of MC-2 ≈ HEC-1.5 > CMC-1.5 >> HPMC-2.

These results highlight significant differences related to network structure and water affinity among the tested cellulose derivatives, which may influence drug release behavior and topical performance.

#### 2.1.5. Weight Loss Results

**Figure 4:**
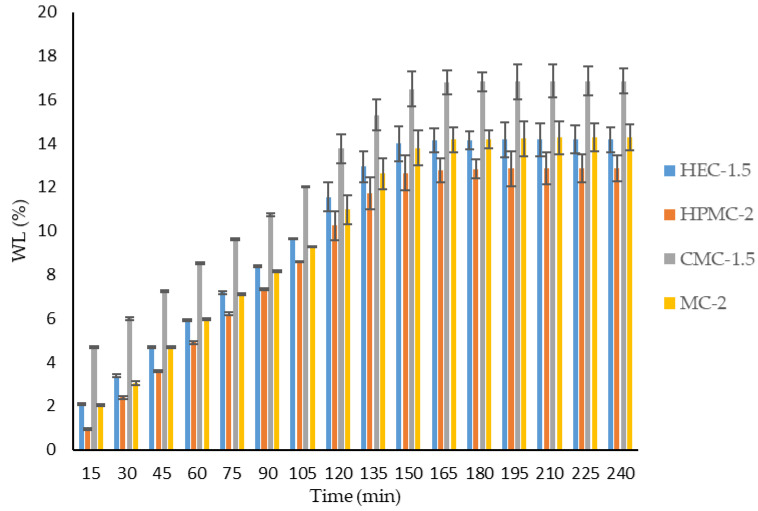
Weight loss (*WL*, %) of cellulose-based hydrogels (HEC-1.5, HPMC-2, CMC-1.5, and MC-2) as a function of time (15–240 min) at 25 ± 0.5 °C. Values are expressed as means ± SDs (n = 3).

The dehydration kinetics of the cellulose-based hydrogels (HEC-1.5, HPMC-2, CMC-1.5, and MC-2) were evaluated over 240 min at 25 ± 0.5 °C. The percentage of weight loss (*WL*, %) as a function of time is shown in Figure 4. All formulations exhibited a progressive increase in weight loss during the first 150–165 min, followed by stabilization, indicating the attainment of equilibrium.

CMC-1.5 displayed the highest dehydration extent, reaching a plateau value of 16.85 ± 0.65% at 240 min. MC-2 and HEC-1.5 showed a comparable behavior, with final weight loss values of 14.27 ± 0.55% and 14.18 ± 0.61%, respectively. In contrast, HPMC-2 demonstrated the lowest water loss, which stabilized at 12.86 ± 0.56%.

The initial phase (15–105 min) was characterized by a nearly linear increase in *WL* (%), reflecting rapid evaporation of loosely bound water. After 120 min, the rate of water loss decreased, suggesting the removal of more strongly retained water associated with polymer–water interactions.

The observed differences in dehydration behavior can be attributed to the intrinsic physicochemical properties of the cellulose derivatives. CMC-1.5 exhibited the highest weight loss, likely due to its higher hydrophilicity and ionic character, which promote greater initial water uptake and subsequent evaporation. Conversely, HPMC-2 showed the lowest dehydration extent, indicating stronger water retention capacity that is possibly related to hydrophobic methoxy substitutions that limit rapid water diffusion.

HEC-1.5 and MC-2 demonstrated intermediate behavior, suggesting a balance between hydrophilic interactions and network compactness. The plateau observed after approximately 165–180 min indicates an equilibrium moisture content under the tested conditions. From a topical application perspective, moderate water loss is desirable to ensure adequate skin residence time while avoiding excessive dehydration that could compromise gel integrity. The results seem to indicate that all formulations maintain structural stability during dehydration, with HPMC-2 exhibiting the highest water retention and CMC-1.5 exhibiting the highest evaporation rate. Overall, the dehydration profiles highlight how polymer chemistry significantly influences hydrogel stability and water retention performance, which are critical parameters for dermal formulations.

### 2.2. Antioxidant and Antibacterial Activity

The antioxidant capacity of MD2- and RO1-based hydrogels was assessed by the DPPH radical scavenging assay and reported as antioxidant activity percentages (*AA*%), as shown in Table 2.

Overall, MD2 formulations showed lower DPPH inhibition values compared to the corresponding RO1 hydrogels. Specifically, MD2-based hydrogels exhibited DPPH values ranging from 16.66% to 24.73%, whereas RO1 formulations showed markedly higher radical scavenging activity, ranging from 64.28% to 71.02%. A similar trend was observed for the extracts alone, with the RO1 extract (65.39 ± 0.22%) displaying higher DPPH activity than the MD2 extract (17.56 ± 0.34%).

Conversely, when antioxidant activity was expressed as ascorbic acid equivalents (*AA*%), MD2 formulations showed higher values (75.28–83.34%) compared to RO1 hydrogels (16.66–31.93%), reflecting the inverse relationship between %DPPH inhibition and residual radical concentration.

Notably, statistical analysis indicated that the combination of polymer matrices with the respective extracts (MD2 and RO1 hydrogels; COMBO) did not result in a statistically significant difference in antioxidant performance when compared to their corresponding extracts alone (*p* > 0.05). This suggests that their incorporation into the hydrogel matrix did not significantly alter the intrinsic antioxidant activity of the extracts, indicating good compatibility between the polymeric systems and the bioactive compounds.

These findings demonstrate that both MD2 and RO1 formulations preserve the antioxidant properties of the incorporated extracts, with no significant enhancement or reduction attributable to the hydrogel matrix.

The minimum inhibitory concentrations (MICs) of the OMW fractions (MD2, RO1, and their combination, COMBO) and their corresponding cellulose-based hydrogel formulations are reported in Table 3. Overall, the purified OMW fractions exhibited relevant antimicrobial activity against both Gram-positive and Gram-negative clinical isolates, including antibiotic-resistant strains.

Among the tested samples, MD2 showed the strongest antibacterial effect, with MIC values of 8 mg/mL against *Staphylococcus aureus*, *Staphylococcus epidermidis*, *Enterococcus faecalis*, and *Enterococcus faecium* and 16 mg/mL against *Escherichia coli* and *Pseudomonas aeruginosa*. Incorporation into cellulose-based hydrogels resulted in a moderate increase in MIC values (generally 16–31 mg/mL), indicating partial attenuation of activity, although antimicrobial efficacy was retained across all tested strains.

RO1 exhibited MIC values ranging from 16 to 31 mg/mL, with slightly reduced activity against certain Gram-negative isolates. Similar trends were observed after formulation into hydrogels, where MIC values were predominantly 31 mg/mL. The combined fraction (COMBO) demonstrated consistent inhibitory activity (16 mg/mL) against most strains, including multidrug-resistant isolates. Notably, the antimicrobial activity of COMBO was largely preserved after incorporation into hydrogels, although some formulations showed increased MIC values (31 mg/mL). 

Statistical analysis of MIC values revealed a significant increase (*p* < 0.05) in MICs after incorporation into the polymeric matrices compared to the corresponding free OMW fractions. However, no statistically significant differences were observed among the different cellulose-based hydrogels (HEC, HPMC, MC, and CMC), suggesting that the type of cellulose derivative did not markedly influence the antimicrobial performance.

The observed increase in MIC values after hydrogel incorporation may be attributed to the diffusion limitations of the bioactive compounds within the polymeric network. In hydrogel systems, phenolic molecules may be partially retained or interact with the polymer chains, resulting in a slower release and reduced immediate availability in the surrounding medium during MIC determination. Consequently, higher apparent MIC values may be measured, even though the intrinsic antimicrobial activity of the OMW fractions remains preserved.

The results demonstrate that all formulations maintained inhibitory activity against clinically relevant antibiotic-resistant strains, including MRSA, MRSE, VRE, KPC-producing *E. coli*, and colistin-resistant *P. aeruginosa*, thus supporting the potential of OMW-loaded hydrogels as topical antimicrobial systems.

## 3. Conclusions

In this study, cellulose-based hydrogels incorporating purified olive mill wastewater (OMW) fractions (RO1, MD2, and their combination) were successfully developed and comprehensively characterized for topical antimicrobial applications. The incorporation of 10% (*w*/*w*) OMW into different cellulose derivatives (CMC, HEC, HPMC, and MC) resulted in homogeneous, stable formulations with physicochemical properties suitable for dermal administration.

All OMW-loaded hydrogels exhibited slightly acidic pH values (4.45–4.97), were compatible with the physiological skin pH, and demonstrated pseudoplastic behavior, ensuring both adequate retention at rest and improved spreadability under mechanical stress. Swelling and weight loss studies revealed polymer-dependent differences in water uptake and dehydration kinetics, highlighting the influence of cellulose chemistry on hydrogel structural stability and moisture management.

Notably, the antioxidant activity of the incorporated OMW fractions was preserved within the hydrogel matrices, as confirmed by the DPPH assay, indicating that the polymeric network did not compromise the bioactive potential of the extracts. From a microbiological perspective, the hydrogels maintained inhibitory activity against a broad panel of clinically relevant Gram-positive and Gram-negative strains, such as MRSA, MRSE, VRE, KPC-producing *E. coli* and MDR *P. aeruginosa*, including a colistin-resistant strain. Although a moderate increase in MIC values was observed after incorporation into the hydrogel matrix, antimicrobial efficacy was retained across all tested formulations.

Overall, these findings suggest that cellulose-based hydrogels may represent sustainable and biocompatible carriers for the valorization of OMW-derived bioactive fractions. The developed systems show promise for topical antimicrobial applications and for the management of oxidative stress-related skin conditions.

Future studies should focus on in vivo evaluation, bioadhesion assessment, and controlled release profiling to further elucidate their clinical applicability.

## 4. Materials and Methods

### 4.1. Preparation of Hydrogels

Hydrogels were prepared by dispersing the appropriate amount of polymeric gelling agent (1.5 or 2.0% *w*/*w*) in deionized water under continuous mechanical stirring at room temperature (approximately 25 °C). The polymer was gradually added to the solvent under low-viscosity conditions to promote proper wetting, hydration, and chain solvation while minimizing the formation of aggregates.

Stirring was maintained until complete polymer dispersion and homogeneous gel formation were achieved. The formulations were subsequently evaluated to identify the most suitable systems for the intended topical application.

The gelling agents used were cellulose derivatives, namely carboxymethyl cellulose (CMC, Blanose^®^, Ashland, Wilmington, DE, USA), hydroxypropyl cellulose (HPC, Klucel^®^, Ashland, OR, USA), hydroxyethyl cellulose (HEC, Natrosol^®^, Ashland, OR, USA), and hydroxypropyl methylcellulose (Benecel^®^, Ashland, OR, USA).

In addition to the blank formulations, hydrogels containing 10% (*w*/*w*) olive mill wastewater (OMW) were also prepared by incorporating the appropriate amount of OMW into the polymer dispersion under continuous stirring until homogeneous systems were obtained. The nomenclature of OMW-containing formulations was defined by a prefix indicating the type of olive mill wastewater used (RO1, MD2, or RO1/MD2 (1:1; COMBO)), followed by the polymer abbreviation and its concentration (e.g., RO1-HEC-1.5). Table 4 summarizes the composition of the hydrogel formulations used in this study.

### 4.2. Physicochemical and Technological Characterization of Hydrogels

#### 4.2.1. Organoleptic Evaluation

Hydrogels were visually inspected immediately after preparation and during storage in terms of color, transparency, homogeneity, and the presence of air bubbles or polymer aggregates. The occurrence of phase separation or syneresis phenomena was also recorded.

#### 4.2.2. pH Determination

The pH of each formulation was measured at 25 ± 0.5 °C using a calibrated digital pH meter (Jenway 3510 Standard Digital pH Meter, Jenway, Staffordshire, UK). The electrode was directly immersed into the gel sample. Measurements were performed in triplicate, and results are expressed as means ± standard deviations (SDs).

#### 4.2.3. Rheological Analysis

Rheological measurements were carried out using a rotational viscometer (Viscostar-R, FUNGILAB S.A., Sant Feliu de Llobregat, Spain) equipped with a coaxial cylinder system (bob and cup geometry) and spindle R5. All measurements were performed at 25 ± 0.5 °C. Flow behavior was evaluated by recording shear stress as a function of shear rate over a range of 1–100 s^−1^. Viscosity was determined from the resulting flow curves. All determinations were performed in triplicate.

#### 4.2.4. Swelling Studies

The swelling behavior of the cellulose-based hydrogels was evaluated using a gravimetric method. A predetermined amount of each formulation (3 g) was accurately weighed (*W*_0_) and placed in a suitable container. Samples were then immersed in an excess amount of distilled water and maintained at 25 ± 0.5 °C.

At predetermined time intervals (0.5, 1, 1.5, 2, 2.5, 3, and 3.5 h), the samples were removed, gently blotted with filter paper to remove excess surface liquid, and reweighed (*W_w_*).

The swelling ratio (*SR*, %) was calculated according to Equation (1) [32]:

(1) $$SR\left(\%\right)=\frac{W_{w}-W_{0}}{W_{0}}\times 100$$

where *W*_0_ is the initial weight of the sample and *W_w_* is the wet weight at time *t*.

All experiments were performed in triplicate, and results are expressed as means ± standard deviations (SDs).

#### 4.2.5. Weight Loss Determination

The weight loss of the hydrogels was evaluated to assess water evaporation and formulation stability over time. A predetermined amount of each hydrogel (2.0 g) was accurately weighed (*W*_0_) and stored under controlled conditions (25 ± 0.5 °C).

At predetermined time intervals (every 15 min up to 4 h), samples were reweighed (*W_t_*), and the percentage of weight loss (*WL*, %) was calculated according to Equation (2) [33]:

(2) $$WL\left(\%\right)=\frac{W_{t}-W_{0}}{W_{0}}\times 100$$

where *W*_0_ is the initial weight of the sample and *W_t_* is the weight at time *t*.

All experiments were performed in triplicate, and results are expressed as means ± standard deviations (SDs).

### 4.3. Antioxidant Properties and Microbiological Evaluation

#### 4.3.1. Determination of Antioxidant Activity by the DPPH Assay

The antioxidant activity of the hydrogel containing olive mill wastewater (OMW) was evaluated using the 2,2-diphenyl-1-picrylhydrazyl (DPPH) radical scavenging assay. Briefly, a DPPH solution (0.1 mM) was prepared in methanol and protected from light. An appropriate amount of the hydrogel was diluted in methanol under stirring and centrifuged, if necessary, to obtain a clear supernatant.

An aliquot of the sample solution was mixed with the DPPH solution at a defined ratio (1:1 *v*/*v*) and incubated in the dark at room temperature for 30 min. The decrease in absorbance was measured at 517 nm using a UV–Vis spectrophotometer (Evolution 300, Fischer Scientific, GmbH, Schwerte, Germany). A control sample containing the DPPH solution and solvent without the hydrogel was used as a reference.

Antioxidant activity (*AA*, %) was calculated according to Equation (3) [34]:

(3) $$AA(\%)=\frac{A_{0}-A_{s}}{A_{0}}\times 100$$

where *A*_0_ is the absorbance of the blank DPPH solution and *A_s_* is the absorbance of the sample. All measurements were performed in triplicate (n = 3), and results are expressed as means ± standard deviations (SDs). A calibration curve of a standard antioxidant (Trolox ranging between 20 and 200 mg/L, R2 = 0.9976) was used to express the results as equivalent antioxidant capacity.

#### 4.3.2. Bacterial Strains and Identification

A total of 18 clinical isolates, including Gram-positive and Gram-negative species, were employed in this study. All strains were obtained from the bacterial collection of the School of Medical and Pharmaceutical Sciences, University of Genoa (Italy). Identification was performed using the VITEK^®^ 2 system or matrix-assisted laser desorption/ionization time-of-flight mass spectrometry (MALDI-TOF MS) (bioMérieux, Florence, Italy).

Among the Gram-positive bacteria (n = 12), six isolates belonged to the genus *Enterococcus*, including three vancomycin-resistant *E. faecalis* (VRE) strains and three *E. faecium* (one vancomycin-susceptible and two VRE) strains. Additionally, six *Staphylococcus* isolates were included, consisting of three methicillin-resistant *S. aureus* (MRSA) strains and three methicillin-resistant *S. epidermidis* (MRSE) strains. The Gram-negative group (n = 6) included three multidrug-resistant (MDR) *P. aeruginosa* isolates and three *E. coli* isolates, one of which produced *Klebsiella pneumoniae* carbapenemase (KPC) β-lactamase. Among the *P. aeruginosa* strains, one was also colistin-resistant, while another was isolated from a patient with cystic fibrosis.

#### 4.3.3. Determination of Minimum Inhibitory Concentrations (MICs)

The antimicrobial activity of the OMW samples, their corresponding hydrogel formulation, and the cellulose-based hydrogels used as controls was evaluated by determining the minimum inhibitory concentrations (MICs) using the microdilution method according to the European Committee on Antimicrobial Susceptibility Testing (EUCAST) guidelines (available online: https://www.eucast.org/ast_of_bacteria/ (accessed on 2 March 2026)), as previously described [29]. The MIC assay was evaluated by visual inspection of bacterial growth inhibition in microtiter plates according to EUCAST guidelines. All assays were performed in triplicate, and complete concordance (3/3) was observed across independent experiments.

### 4.4. Statistical Analysis

All experimental measurements were performed at least in triplicate. Data are ex-pressed as means ± standard deviations (SDs). Statistical analysis was performed using [GraphPad Software 8, San Diego, CA, USA]. Differences between formulations were evaluated using one-way ANOVA followed by Tukey’s post hoc test, with *p* < 0.05 considered statistically significant.

## Figures and Tables

**Table 1:** pH values of cellulose-based hydrogels loaded with 10% (*w*/*w*) olive mill wastewater (OMW) fractions (MD2 and RO1). Values are expressed as means (n = 3) ± standard deviations (SDs).

	pH
MD2-HPMC-2	4.95 ± 0.02
RO1-HPMC-2	4.75 ± 0.03
MD2-CMC-1.5	4.97 ± 0.01
RO1-CMC-1.5	4.55 ± 0.04
MD2-HEC-1.5	4.82 ± 0.08
RO1-HEC-1.5	4.63 ± 0.05
MD2-MC-2	4.78 ± 0.02
RO1-MC-2	4.45 ± 0.01

**Table 2:** Antioxidant activity of OMW-loaded hydrogels evaluated by the DPPH assay. Values are expressed as means ± SDs (n = 3).

Sample Code	DPPH (%)	*AA* (%)
MD2-CMC-1.5	16.66 ± 0.58	83.34 ± 0.58
MD2-HPMC-2	24.43 ± 1.67	75.57 ± 1.67
MD2-MC-2	20.29 ± 0.69	79.72 ± 0.69
MD2-HEC-1.5	24.73 ± 2.09	75.28 ± 2.09
RO1-CMC-1.5	64.28 ± 0.24	16.66 ± 0.58
RO1-HPMC-2	71.02 ± 0.55	28.98 ± 0.55
RO1-MC-2	68.07 ± 0.78	31.93 ± 0.78
RO1-HEC-1.5	70.55 ± 1.51	29.45 ± 1.51
MD2	17.56 ± 0.34	82.44 ± 0.35
RO1	65.39 ± 0.22	34.61 ± 0.21

**Table 3:** MIC values expressed as mg/mL of the hydrogels OMW samples on the selected Gram- positive and Gram-negative strains. Experiments were carried out in triplicate.

MIC (mg/mL)																		
Sample Code	*S. aureus*			*S. epidermidis*			*E. faecalis*			*E. faecium*			*E. coli*			*P. aeruginosa*		
	18 *	187 *	188 *	22 ^#^	180 ^#^	222 ^#^	1	50 ^V^	365 ^V^	300 ^V^	362 ^V^	40	224	238 ^K^	4	1V ^A^	265 °	403
**MD2**	8	8	8	8	8	16	16	16	16	16	16	16	16	16	16	16	16	8
**MD2-HEC-1.5**	16	16	16	31	31	31	31	31	31	31	31	31	31	31	31	31	31	31
**MD2-HPMC-2**	16	16	16	31	31	31	31	31	31	31	31	31	31	31	31	31	31	31
**MD2-MC-2**	16	16	16	31	31	31	31	31	31	31	31	31	31	31	31	31	31	31
**MD2-CMC-1.5**	16	16	16	31	31	31	31	31	31	31	31	31	31	31	31	31	31	31
**RO1**	16	31	16	16	16	16	16	16	16	16	16	16	31	31	31	16	16	16
**RO1-HEC-1.5**	16	16	16	16	16	16	16	16	16	31	31	31	31	31	31	31	31	31
**RO1-HPMC-2**	31	31	31	31	31	31	31	31	31	31	31	31	31	31	31	31	31	31
**RO1-MC-2**	31	31	31	31	31	31	31	31	31	31	31	31	31	31	31	31	31	31
**RO1-CMC-1.5**	31	31	31	31	31	31	31	31	31	31	31	31	31	31	31	31	31	31
**COMBO**	16	16	16	16	16	16	16	16	16	16	16	16	16	16	16	16	16	16
**COMBO-HEC-1.5**	16	16	16	16	16	16	16	16	16	31	31	31	31	31	31	16	16	16
**COMBO-HPMC-2**	31	31	31	31	31	31	31	31	31	31	31	31	31	31	31	16	16	16
**COMBO-MC-2**	31	31	31	31	31	31	31	31	31	31	31	31	31	31	31	31	31	31
**COMBO-CMC-1.5**	31	31	31	31	31	31	31	31	31	31	31	31	31	31	31	31	31	31

* indicates resistance to oxacillin; ^#^ indicates resistance to MRSE; ^V^ indicates resistance to vancomycin; ^K^ indicates strain producing *Klebsiella pneumoniae* carbapenemase (KPC) β-lactamase; ^A^ indicates an MDR strain isolated from a patient with cystic fibrosis; ° indicates resistance to colistin.

**Table 4:** Composition of hydrogel formulations based on cellulose derivatives.

Sample Code	Polymer	Concentration (% *w*/*w*)
HEC-1.5	Hydroxyethyl cellulose	1.5
HPMC-2	Hydroxypropyl methylcellulose	2.0
CMC-1.5	Carboxymethyl cellulose	1.5
MC-2	Methylcellulose	2.0

## Data Availability

The data presented in this study are available in the article.
